# Evaluation of focal damage in the retinal pigment epithelium layer in serous retinal pigment epithelium detachment

**DOI:** 10.1038/s41598-019-39688-z

**Published:** 2019-03-01

**Authors:** Masahiro Miura, Shuichi Makita, Shinnosuke Azuma, Yoshiaki Yasuno, Shunichiro Ueda, Satoshi Sugiyama, Toshihiro Mino, Tatsuo Yamaguchi, Harpal S. Sandhu, Henry J. Kaplan, Takuya Iwasaki, Hiroshi Goto

**Affiliations:** 1 Department of Ophthalmology, Tokyo Medical University, Ibaraki Medical Center, Inashiki, Japan; 2 Department of Ophthalmology, Tokyo Medical University, Tokyo, Japan; 3 Computational Optics Group, University of Tsukuba, Tsukuba, Japan; 4 Tomey Corporation, Nagoya, Japan; 5 Topcon Corporation, Tokyo, Japan; 6 Department of Ophthalmology and Visual Sciences, University of Louisville, Louisville, KY USA

## Abstract

The purpose of this study was to evaluate focal damage in the retinal pigment epithelium (RPE) layer in serous retinal pigment epithelium detachment (PED) with multi-contrast optical coherence tomography (OCT), which is capable of simultaneous measurement of OCT angiography, polarization-sensitive OCT and standard OCT images. We evaluated 37 eyes with age-related macular degeneration that had serous PED. Focal RPE damage was indicated by hyper-transmission beneath the RPE-Bruch’s membrane band in standard OCT images. Distribution of RPE melanin was calculated using the dataset from multi-contrast OCT. Twenty-four points with hyper-transmission were detected in 21 of the 37 eyes. Standard OCT images failed to show disruption of the RPE-Bruch’s membrane band at 5 of the 24 hyper-transmission points. Conversely, multi-contrast OCT images clearly showed melanin defects in the RPE-Bruch’s membrane band at all points. Areas of melanin defects with disruption of the RPE-Bruch’s membrane band were significantly larger than those without disruption. The volume of intraretinal hyper-reflective foci was significantly larger in eyes with hyper-transmission than that in eyes without hyper-transmission. Multi-contrast OCT is more sensitive than standard OCT for displaying changes at the RPE-Bruch’s membrane band when there are small areas of RPE damage.

## Introduction

Age-related macular degeneration (AMD) remains a leading cause of severe vision loss in most developed countries^1^. Pathological changes in the retinal pigment epithelium (RPE) are important indicators of AMD development, and early detection of RPE changes is important in the evaluation of AMD^2^. Retinal pigment epithelium detachment (PED) is a frequent finding associated with the disease^3^. Recently, focal RPE damage in serous PEDs has been identified as a precursor to the shrinkage or collapse of PEDs or the development of RPE tears, all of which are clinically important causes of vision loss^3,4^. Clinically, focal RPE damage in PEDs can be detected as a zone of attenuation or disruption of the RPE-Bruch’s membrane band, with hyper-transmission in the choroid in intensity-based optical coherence tomography (OCT) B-scan images: so-called standard OCT B-scan images^4^. Previous studies showed that the RPE-Bruch’s membrane band in standard OCT images may persist to some extent, despite focal RPE damage, owing to a lack of specific contrast in RPE cells^5,6^. This essential limitation of standard OCT might impede the clinical study of RPE damage in AMD.

Polarization-sensitive OCT (PS-OCT) is a functional extension of OCT technology that acquires three-dimensional (3D) polarization images of the eye^7,8^. Melanin in tissues, including the RPE and choroid, can scatter light, causing depolarization or polarization scramble^9^. Multiple studies have established that PS-OCT can detect RPE changes^7,8^. However, depolarized retinal images show both choroidal^10^ and RPE^11,12^ melanin; hence, separate extraction of RPE melanin is important for proper evaluation of RPE changes without the influence of choroidal melanin. Our group developed a pixel-wise segmentation method for RPE melanin using multi-contrast OCT (MC-OCT), and analyzed RPE-melanin cross-sectional images to simplify the evaluation of RPE damage^13^. RPE-melanin cross-sectional images might be useful for evaluating focal RPE damage in PEDs. In this study, we compared the sensitivity of MC-OCT to standard OCT for revealing focal RPE damage in PEDs, and we correlated the MC-OCT findings with other clinical findings in AMD.

## Results

**Figure 1:**
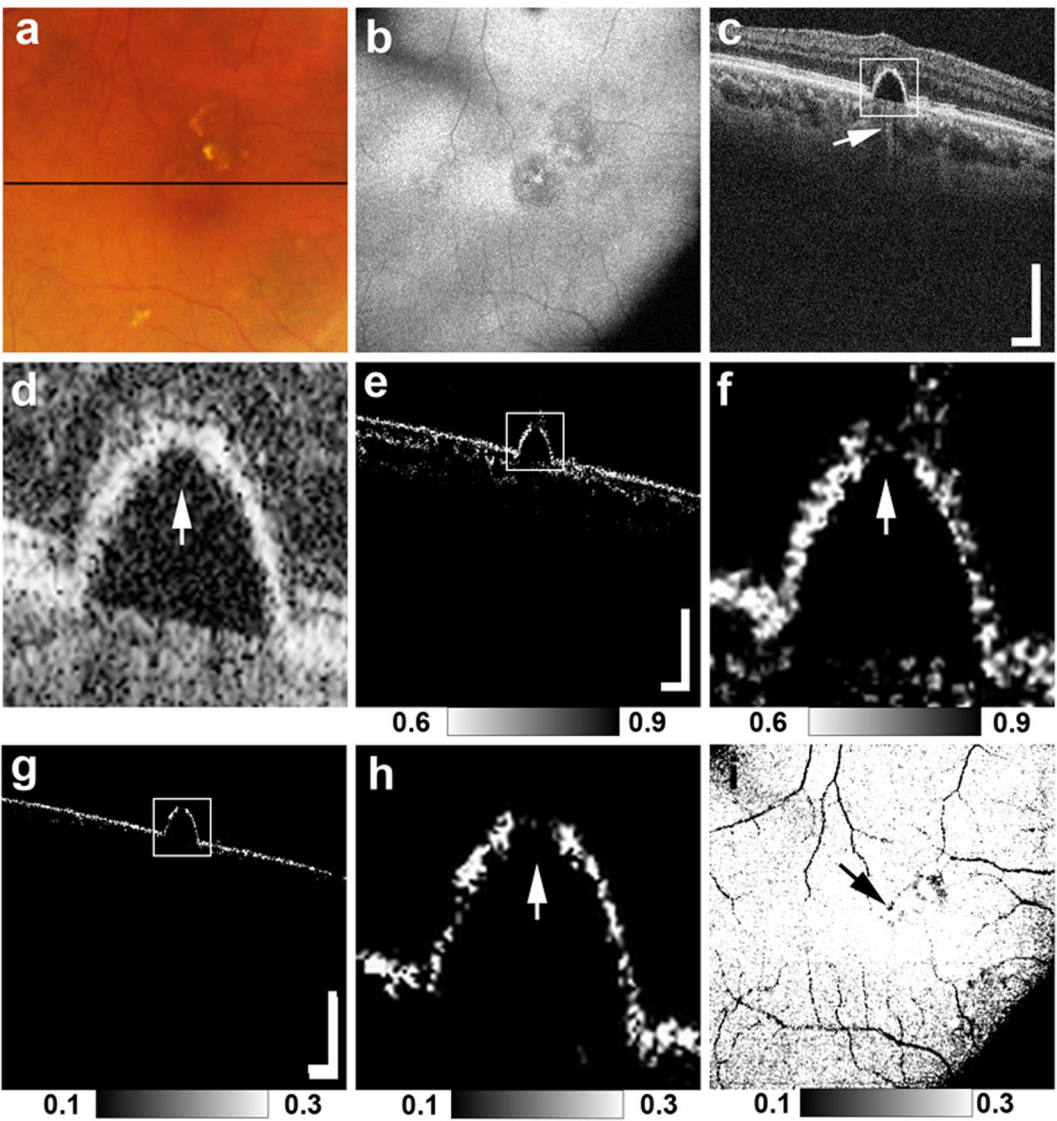
MC-OCT imaging of focal RPE damage classified into pattern 1 in the right eye of an 80-year-old male. The black line in the color fundus photograph (**a**) indicates the scan line of the MC-OCT. The *en face* projection of the standard OCT image (**b**) The white squares in the B-scan images (**c**,**e**,**g**) show the areas of the high-magnification images (**d**,**f**,**h**) The standard OCT B-scan images (**c**,**d**) show hyper-transmission beneath the RPE-Bruch’s membrane band (**c**: white arrow) without attenuation or disruption in the RPE-Bruch’s membrane band at the corresponding location (**d**: white arrow). A DOPU B-scan image (**e**,**f**) shows the defect of depolarization at the RPE-Bruch’s membrane band (f: white arrow). RPE-melanin cross-sectional images (**g**,**h**) clearly show an RPE melanin defect at the RPE-Bruch’s membrane band (h: white arrow). The *en face* projection of the maximum F_RPE_ (**i**) shows the low-intensity area at the point of focal RPE damage (black arrow). The scale bars represent 500 μm × 500 μm.

**Figure 2:**
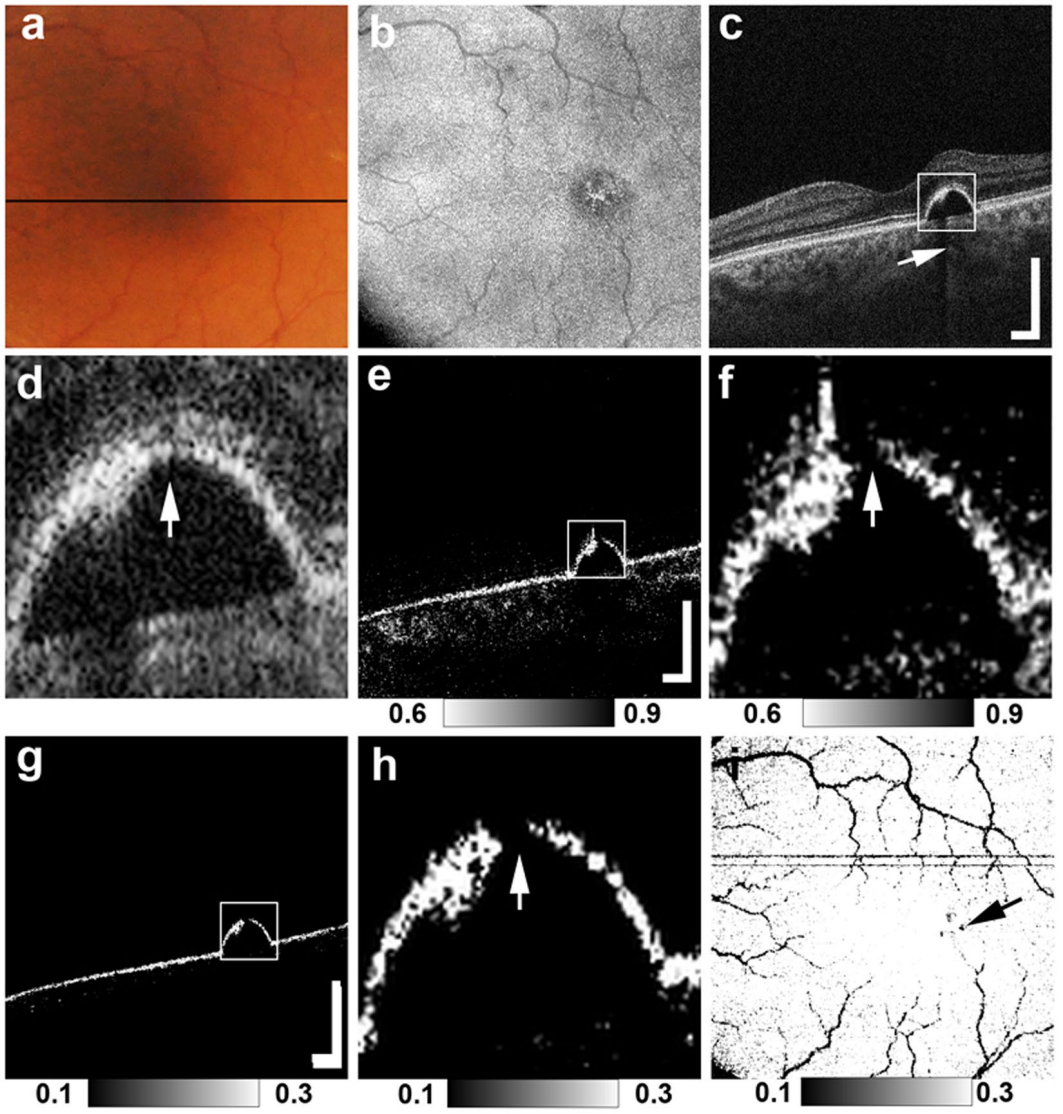
MC-OCT imaging of focal RPE damage classified into pattern 2 in the left eye of a 65-year-old female. The black line in the color fundus photograph (**a**) indicates the scan line of the MC-OCT. The *en face* projection of the standard OCT image (**b**). The white squares in the B-scan images (**c**,**e**,**g**) show the areas of the high-magnification images (**d**,**f**,**h**). The standard OCT B-scan images (**c**,**d**) show hyper-transmission beneath the RPE-Bruch’s membrane band (**c**: white arrow) with mild attenuation in the RPE-Bruch’s membrane band at the corresponding location (d: white arrow). DOPU B-scan images (**e**,**f**) and RPE-melanin cross-sectional images (**g**,**h**) show the focal RPE change at the RPE-Bruch’s membrane band (**f**,**h**: white arrows). The *en face* projection of the maximum F_RPE_ (**i**) shows the low-intensity area at the point of focal RPE damage (black arrow). The scale bars represent 500 μm × 500 μm.

**Figure 3:**
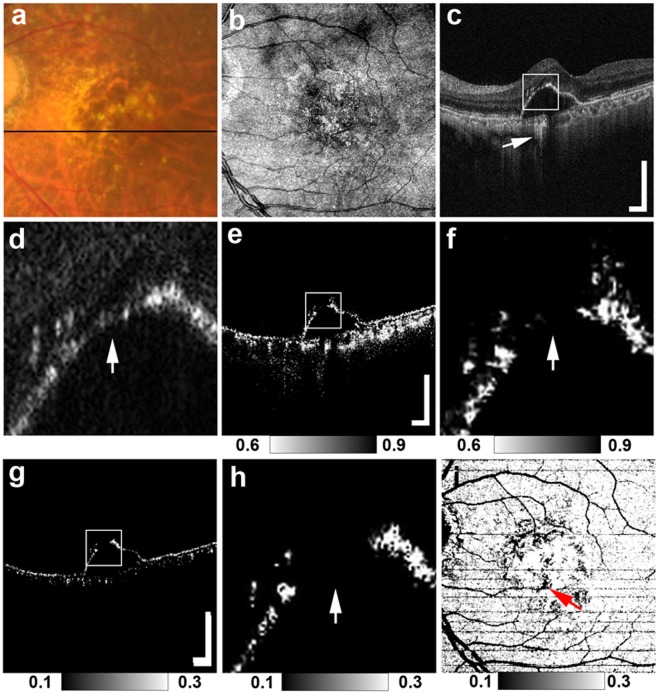
MC-OCT imaging of focal RPE damage classified into pattern 3 in the left eye of a 75-year-old female. The black line in the color fundus photograph (**a**) indicates the scan line of the MC-OCT. The *en face* projection of the standard OCT image (**b**). The white squares in the B-scan images (**c**,**e**,**g**) show the areas of the high-magnification images (**d**,**f**,**h**). The standard OCT B-scan images (**c**,**d**) show hyper-transmission beneath the RPE-Bruch’s membrane band (**c**: white arrow) with clear attenuation and disruption in the RPE-Bruch’s membrane band at the corresponding location (**d**: white arrow). DOPU B-scan images (**e**,**f**) and RPE-melanin cross-sectional images (**g**,**h**) show the focal RPE change at the RPE-Bruch’s membrane band (**f**,**h**: white arrows). The *en face* projection of the maximum F_RPE_ (**i**) shows the low-intensity area at the point of focal RPE damage (red arrow). The scale bars represent 500 μm × 500 μm.

A total of 24 points of hyper-transmission beneath the RPE-Bruch’s membrane were detected in 21 (57%) of 37 eyes in standard OCT B-scan images. Two points of hyper-transmission were detected in each of three eyes. In each of the other eyes with hyper-transmission, only a single point of hyper-transmission was detected. RPE-melanin cross-sectional images, which were obtained with MC-OCT, clearly showed the defect of RPE melanin at all points with hyper-transmission (Figs 1, 2 and 3). For each point of hyper-transmission, two ophthalmologists (M.M. and S.U.) subjectively rank-ordered the presence of attenuation or disruption at the RPE-Bruch’s membrane band in standard OCT images.

Pattern 1: RPE-Bruch’s membrane band appeared continuous, without attenuation or disruption.

Pattern 2: RPE-Bruch’s membrane band showed mild attenuation or tiny disruption

Pattern 3: RPE-Bruch’s membrane band was faded with clear disruption.

In the case of discrepancies in specifying the pattern, a third observer (I.T.) acted as a referee and reached a consensus.

By the subjective evaluation of the RPE-Bruch’s membrane band at hyper-transmission points in standard OCT images, 5 points were classified as pattern 1, 10 points were classified as pattern 2, and 9 points were classified as pattern 3. The weighted Kappa value of inter-observer agreement (M.M. and S.U.) was 0.75.

**Figure 4:**
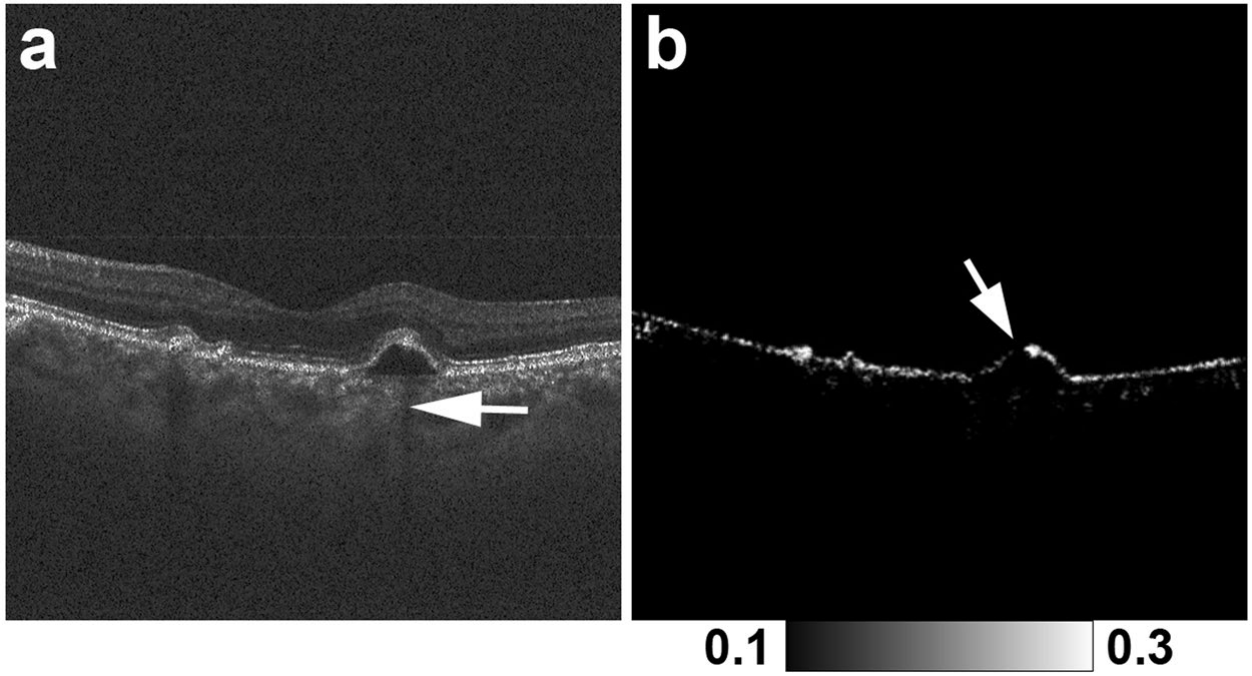
MC-OCT imaging of focal RPE damage classified into pattern 2 in the left eye of a 70-year-old female shows fuzzy hyper-transmission (white arrow) beneath the RPE-Bruch’s membrane band in a standard OCT B-scan image (**a**). RPE-melanin cross-sectional images (**b**) show an RPE melanin defect at the RPE-Bruch’s membrane band (white arrow).

Figures 1, 2 and 3 show representative cases of patterns 1, 2 and 3, respectively. The color fundus photographs, as typically seen in clinical practice, showed the lateral location of the lesions but provided no 3D information about the focal RPE damage (Figs 1a, 2a and 3a). Similarly, the computed *en face* projection images from standard OCT imaging lacked the depth information needed to detect focal RPE damage (Figs 1b, 2b and 3b). The standard OCT B-scan images were retinal cross-sections that documented the presence of hyper-transmission beneath the RPE-Bruch’s membrane band. Findings at the RPE-Bruch’s membrane band in patterns 1, 2 and 3 were clearer in a stepwise fashion (Figs 1d, 2d and 3d). At all of the points of hyper-transmission, the external limiting membrane band was visible; however the ellipsoid zone band was interrupted in standard OCT images. In PS-OCT, the depolarization of the tissue can be evaluated by using a quantity so called as degree of polarization uniformity (DOPU)^7,8^. DOPU is a circular variance of polarization states of the back scattered OCT probe beam computed on Poincaré sphere and it represents randomness of the polarization. DOPU B-scan OCT images in PS-OCT showed depolarization consistent with RPE changes and the focal RPE melanin defects (Figs 1f, 2f and 3f). However, the RPE melanin defects in DOPU B-scan images were somewhat fuzzy for small RPE defects (Fig. 1f). We developed an index, termed F_RPE_, for automatic detection of RPE melanin using DOPU, the OCT attenuation coefficient, and OCT angiography (see the Methods section for details)^13^. We calculated the RPE-melanin cross-sectional images that represent the distribution of F_RPE_ in the B-scan images. RPE-melanin cross-sectional images clearly showed an RPE melanin defect at all hyper-transmission points (Figs 1h, 2h and 3h). The clarity of the hyper-transmission in standard OCT images varied widely among the cases. In some eyes with small amounts of RPE damage in pattern 1 or 2, hyper-transmission was somewhat fuzzy (Fig. 4). However, RPE-melanin cross-sectional images clearly showed the RPE melanin defect even at these fuzzy hyper-transmission points. In addition, we calculated *en face* projections of the maximum F_RPE_ values. These maps showed the low intensity of RPE-melanin at points of focal RPE damage (Figs 1i, 2i and 3i).

**Figure 5:**
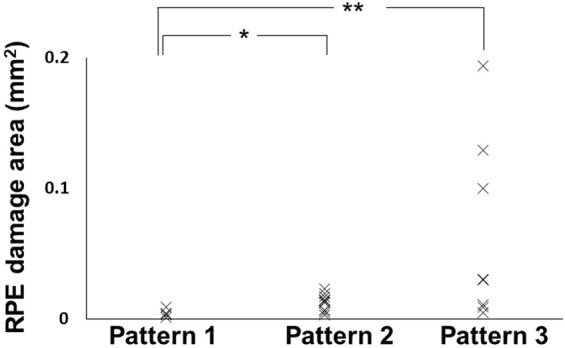
Distribution of sizes of the regions of focal RPE damage with patterns 1, 2 and 3. (*P = 0.029, **P = 0.003, Mann-Whitney U test).

We compared the mean area of focal RPE melanin defects in MC-OCT images among patterns 1, 2 and 3 (Fig. 5). The mean areas of focal RPE melanin defects were 0.004 mm^2^ (5 points, standard deviation (SD): 0.003 mm^2^, range: 0.001–0.009 mm^2^), 0.012 mm^2^ (10 points, SD: 0.006 mm^2^, range: 0.003–0.029 mm^2^), 0.064 mm^2^ (9 points, SD: 0.070 mm^2^, range: 0.005–0.194 mm^2^) in patterns 1, 2 and 3, respectively. The mean areas of focal RPE melanin defects in patterns 2 and 3 were significantly larger than in pattern 1 (P = 0.029 and 0.003, respectively, Mann-Whitney U test). There was no significant difference in area between patterns 2 and 3 (P = 0.091, Mann-Whitney U test).

**Figure 6:**
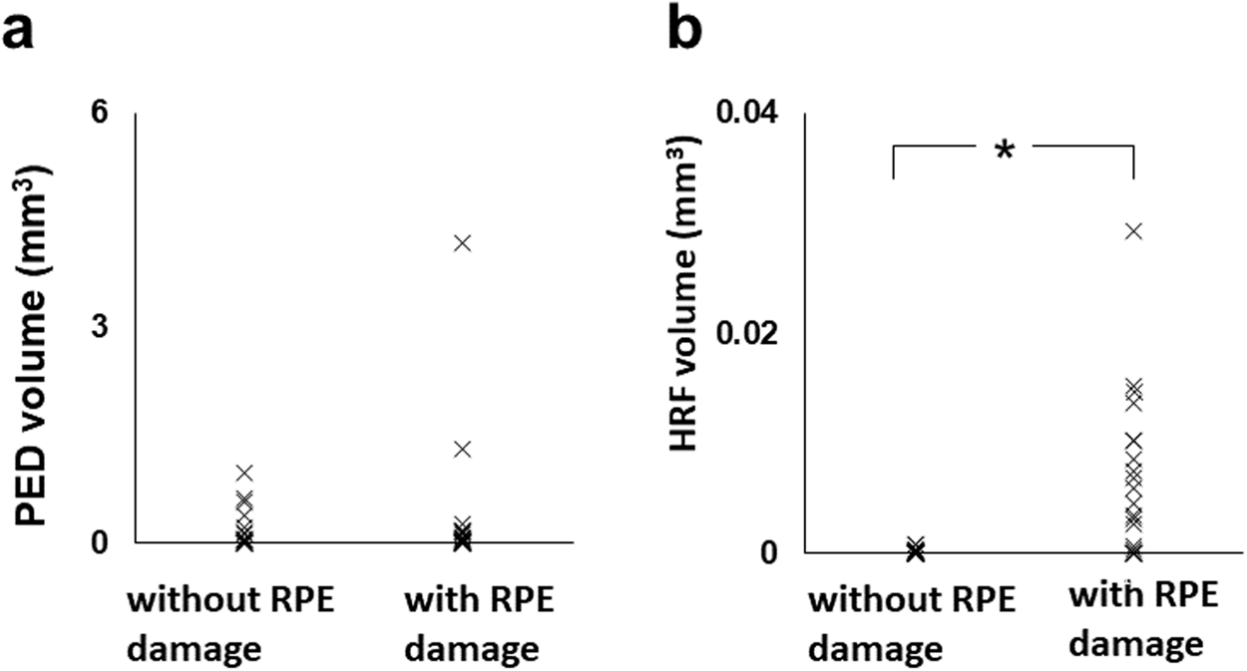
Scatterplots of PED volume and HRF volume in relation to the presence of focal RPE damage. (**a**) Distribution of the PED volumes in the eyes with and without focal RPE damage. (**b**) Distribution of the HRF volume in the eyes with and without focal RPE damage. (*P < 0.001, Mann-Whitney U test).

To evaluate the association of focal RPE damage with other clinical parameters, we compared its presence with the PED volume and intraretinal hyper-reflective foci (HRF) volume in PEDs (Fig. 6). Mean PED volumes were 0.324 mm^3^ (21 eyes, SD: 0.926 mm^3^, range: 0.002–4.178 mm^3^) and 0.199 mm^3^ (16 eyes, SD: 0.287 mm^3^, range: 0.002–0.964 mm^3^) in eyes with and without focal RPE damage, respectively. There was no significant difference in PED volumes based on the presence or absence of focal damage (P = 0.84, Mann-Whitney U test). Mean HRF volumes were 0.0065 mm^3^ (21 eyes, SD: 0.0073 mm^3^, range: 0.0000–0.0294 mm^3^) and 0.0002 mm^3^ (16 eyes, SD: 0.0003 mm^3^, range: 0.0000–0.0009 mm^3^) in the eyes with and without focal RPE damage, respectively. Mean HRF volume was significantly larger for eyes with focal RPE damage than for those without focal RPE damage (P = 0.001, Mann-Whitney U test).

## Discussion

Focal RPE damage has been reported as an important finding in the lifecycle of serous PEDs^4^. In the present study, we evaluated areas of focal RPE damage with the concurrent confirmation of hyper-transmission in standard OCT imaging and RPE melanin loss in MC-OCT imaging. Our results showed that focal RPE damage was frequently present in serous PEDs, and that it was associated with HRF in serous PEDs.

Recently, the Classification of Atrophy Meeting Group proposed an OCT-based definition of RPE atrophy^14^. In their proposition, complete RPE atrophy was defined by concurrent findings: (1) a region of hyper-transmission at least 250 μm in diameter, (2) a zone of attenuation or disruption of the RPE at least 250 μm in diameter, and (3) the absence of scrolled RPE or other signs of an RPE tear. Although this definition could be applied for a certain size of RPE atrophy, it might not be applicable for small areas of early RPE damage. Previous PS-OCT studies have shown that standard OCT can miss some areas of RPE damage^5,6^. In our study, we compared standard OCT images to MC-OCT images, and showed that attenuation or disruption of the RPE band at points of focal RPE damage was frequently not detectable (5 of 24 points; 21%) on standard OCT images. Furthermore, visualization of hyper-transmission may be fuzzy in the early stage of RPE damage^15^, and is influenced by signal attenuation in the choroid. With the shadow effect of concurrent lesions, detection of hyper-transmission might be more complicated. Thus, because of the possibility of missing focal RPE damage, a clinical evaluation of RPE change in AMD, using standard OCT, could produce incomplete findings. However, the present study shows the superior efficacy of MC-OCT for the depiction of small areas of focal RPE damage.

There are several possible reasons for relatively low detectability of small areas of RPE damage in standard OCT images. One possible reason is reduced melanosomes in RPE cells. One histopathological study showed that degranulation of melanolipofuscin occurred in the early stage of RPE damage, and might be a source of hypo-autofluorescence in clinical retinal autofluorescence imaging^16^. Even after the reduction of RPE melanin, OCT signals at RPE-Bruch’s membrane band might be maintained by the presence of phagosomes and mitochondria in RPE cells^17^. Another possibility is the configurational change of RPE cells and deposition of inflammatory cells. Sloughed RPE cells are common finding in AMD^2^. Inflammatory cells were reported to be present in various stages of AMD^18,19^. The sloughed RPE cells and inflammatory cells might appear as focal thickened RPE-Bruch’s membrane band in standard OCT images^2^, and might hinder the detection of the attenuation of RPE-Bruch’s membrane band at points of focal RPE damage. The resolution of our OCT system might be insufficient to detect these cellular-level changes. Comparisons with histopathology are important to confirm these possible findings. Higher-resolution imaging systems with adaptive optics might improve detectability in intensity-based standard OCT^15,20^.

In this study, we found that focal RPE damage was related to HRF. HRF are important OCT findings in AMD because of the association of HRF with the development of geographic atrophy^21–24^. In previous studies, several possibilities concerning the origins of HRF were reported, including lipoprotein aggregation^25^, inflammatory cells^26^, and RPE migration^22,27,28^. Our group previously evaluated HRF in AMD with multimodal imaging and we reported that intraretinal RPE migration was a major source of HRF in serous PEDs^28^. Histopathology confirmed the intraretinal RPE migration at HRF in some type of AMD^27,29^. Intraretinal RPE migration in AMD has been reported as a precursor to chorioretinal atrophy in the death pathway of RPE cells^2^. This study underscores the importance of HRF as a relevant finding associated with focal RPE damage.

**Figure 7:**
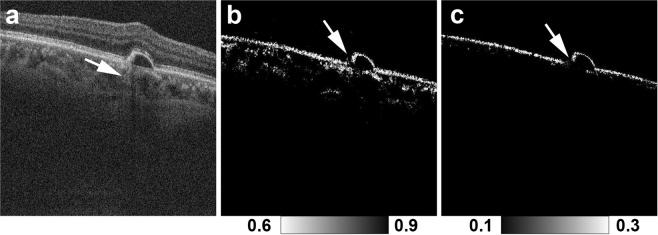
Focal RPE damage at the margin of the PED in the right eye of an 80-year-old male. The standard OCT B-scan image (**a**) shows hyper-transmission at the margin of the PED (white arrow). In the DOPU B-scan image (**b**), adjacent choroidal melanin impedes the detection of RPE damage at the margin of the PED (white arrow). The RPE-melanin cross-sectional image (**c**) clearly shows the RPE melanin defect at the margin of the PED (white arrow).

Previously, Schütze *et al*. demonstrated the use of PS-OCT for detecting areas of focal RPE damage in AMD. In their study, DOPU was used to evaluate RPE melanin^5,6^. They reported that standard OCT might miss some areas of focal RPE damage that can be detected with PS-OCT^5,6^. This study confirms and elaborates upon those findings. This study used RPE-melanin cross-sectional images to simplify the evaluation of RPE damage. In PS-OCT, depolarization caused by melanin appeared in both the RPE and choroid. If RPE damage occurred at the margin of a PED, then adjacent choroidal melanin might impede the evaluation of RPE melanin (Fig. 7)^5,6^. Ethnic variations in the density of choroidal melanin^30^ and large variations of depolarization at the choroid in PS-OCT images^10^ have been reported. Variations in choroidal melanin might influence the evaluation of RPE damage with DOPU. Instead, our study jointly used OCT attenuation information, OCT angiography and DOPU to remove the influence of choroidal melanin, so that RPE damage could readily be detected even at the margin of PEDs (Fig. 7). Moreover, Schütze *et al*. evaluated the detectability of focal RPE damage in a descriptive fashion without observing hyper-transmission in standard OCT images^5,6^, but the present study comprehensively confirms MC-OCT’s superior accuracy when used to detect focal areas of RPE damage. The interpretation of PS-OCT findings remains under discussion; hence, concurrent confirmation of MC-OCT findings with hyper-transmission in standard OCT is important.

Given these strengths, our study substantiates the utility of MC-OCT in the evaluation of AMD. This study has several limitations. It did not employ histopathological analysis of the areas of focal RPE damage. Thus, we cannot confirm our hypothesis about the preservation of the RPE-Bruch’s membrane band seen on standard OCT imaging at points of focal RPE damage. Furthermore, our study could not confirm the origin of HRF. Further studies using histopathology are required to answer these outstanding questions. This study was based on the detection utility for depolarization at the RPE melanin with PS-OCT. In previous PS-OCT studies, depolarization could be detected at the RPE-Bruch’s membrane band, but not at the interdigitation zone band^11,12^. Histological study shows numerous melanosomes in the apical process of RPE cells at the interdigitation zone^31,32^, hence some melanosomes in RPE cells might not be detected with PS-OCT. Further studies are required for the interpretation of polarimetry findings. In this study, a certain number of the threshold of F_RPE_ (≥0.15) in MC-OCT imaging was used to calculate the area of RPE melanin defects, by reference to our previous study^13^. Further study with concomitant histopathology is required to determine the appropriate threshold for evaluating focal RPE damage. Furthermore, with the small number of patients in each pattern group, this study evaluated only some aspects of focal RPE damage. In addition, this study involved only evaluations of serous PEDs. Evaluation of other subtypes of AMD is important for the comprehensive investigation of RPE damage in AMD.

In conclusion, this study demonstrated the clinical utility of MC-OCT imaging in evaluating focal RPE damage in AMD. MC-OCT is more sensitive than standard OCT for the detection of changes at the RPE-Bruch’s membrane band in small areas of focal RPE damage. As this technology improves in the future, MC-OCT has the potential to function in isolation, following technical development. Furthermore, MC-OCT can simultaneously provide standard OCT images; hence, replacement of commercial standard OCT machines would not be difficult. MC-OCT is therefore an effective tool for characterizing RPE changes in macular disease.

## Methods

### Subjects

We prospectively examined 37 eyes of 31 Japanese patients with serous PEDs due to AMD (23 males, 8 females; age range, 45–94 years; mean age, 67.7 years). The term “serous PED” was applied to cases of serous PED without retinal or subretinal hemorrhage. OCT angiography images obtained from MC-OCT were used to exclude eyes with choroidal neovascularization. Eyes with severe cataract or other eye diseases that could significantly compromise the image quality were excluded from this study.

This cross-sectional study adhered to the tenets of the Declaration of Helsinki and was approved by the Institutional Review Boards of Tokyo Medical University, Ibaraki Medical Center (IRB 16–15). The study was registered in a public database (UMIN000026307; http://www.umin.ac.jp/ctr/index-j.htm). The nature of the current study and the implications of participating in this research were explained to all study candidates, and written informed consent was obtained from each participant before any study procedures or examinations were performed.

### MC-OCT imaging

A research prototype of Jones matrix-based MC-OCT^33^ and its simplified system^34^ were used to obtain standard OCT, OCT angiography and degree of polarization uniformity (DOPU) images. The depth resolutions in tissue were 6.6 μm and 6.0 μm, and the lateral resolutions at the retina were 32 μm and 20 μm, for the MC-OCT prototype and the simplified system, respectively. These MC-OCT systems used a swept-source laser with a central wavelength of 1048 nm and an axial scan speed of 100,000 A-scans/second. A horizontal-fast raster scanning protocol with 512 A-lines × 256 B-scans covering a 6.0 × 6.0 mm region on the retina was used for volumetric scans. B-scan measurements were repeated four times at a single location. The acquisition duration of each volumetric measurement was 6.6 seconds. MC-OCT volumes without significant motion artifacts were used for this study.

OCT angiography was computed by the complex Jones matrix correlation method with noise correction^35^. The DOPU was calculated to evaluate the depolarization or polarization scramble of the tissue^36^. Points of low DOPU represent depolarization by multiple scattered lights from melanosomes. In our analyses, the DOPU with Makita’s noise correction was computed using a 3 pixel (transverse) × 3 pixel (depth) kernel^37^. Standard B-scan OCT images were obtained by coherent composition of four repetitive B-scans. Such a standard OCT image approximately corresponds to a B-scan image on a commercial swept-source OCT with a 1-μm-band light source.

We derived a new index which is specific to melanin in the RPE (RPE-melanin) from MC-OCT images and used it for automatic segmentation of the RPE-melanin^13^. Melanin in both the RPE and choroidal stroma showed low DOPU in PS-OCT, with high reflectivity in intensity-based OCT. The choroidal lumen showed low reflectivity in intensity-based OCT. However, the blood flow signal in OCT angiography at the RPE-melanin layer is low due to the absence of vascularization, whereas the choroidal stroma and lumen showed high blood flow signal due to dense vasculature. We calculated a new index (F_RPE_) which is specific to RPE-melanin:

$${{\rm{F}}}_{{\rm{RPE}}}=\mathrm{attenuation}\,{\rm{coefficient}}\times (1-{\rm{DOPU}})\times (1-{{\rm{OCTA}}}_{{\rm{b}}})$$

where the attenuation coefficient is computed with the algorithm presented by Vermeer *et al*.^38^ and is represented as the logarithm (base 10) of the attenuation coefficient in mm^−1^. “OCTA_b_” is the binarized OCT angiography signal obtained by applying Ohtsu’s method^39^ to the raw OCT angiography images. The cross-sectional view of the RPE-melanin specific contrast is used to show the depth-resolved distribution of RPE melanin. An *en face* projection of the maximum F_RPE_ was created to evaluate the *en face* distribution of F_RPE_.

### Evaluation of focal RPE damage

**Figure 8:**
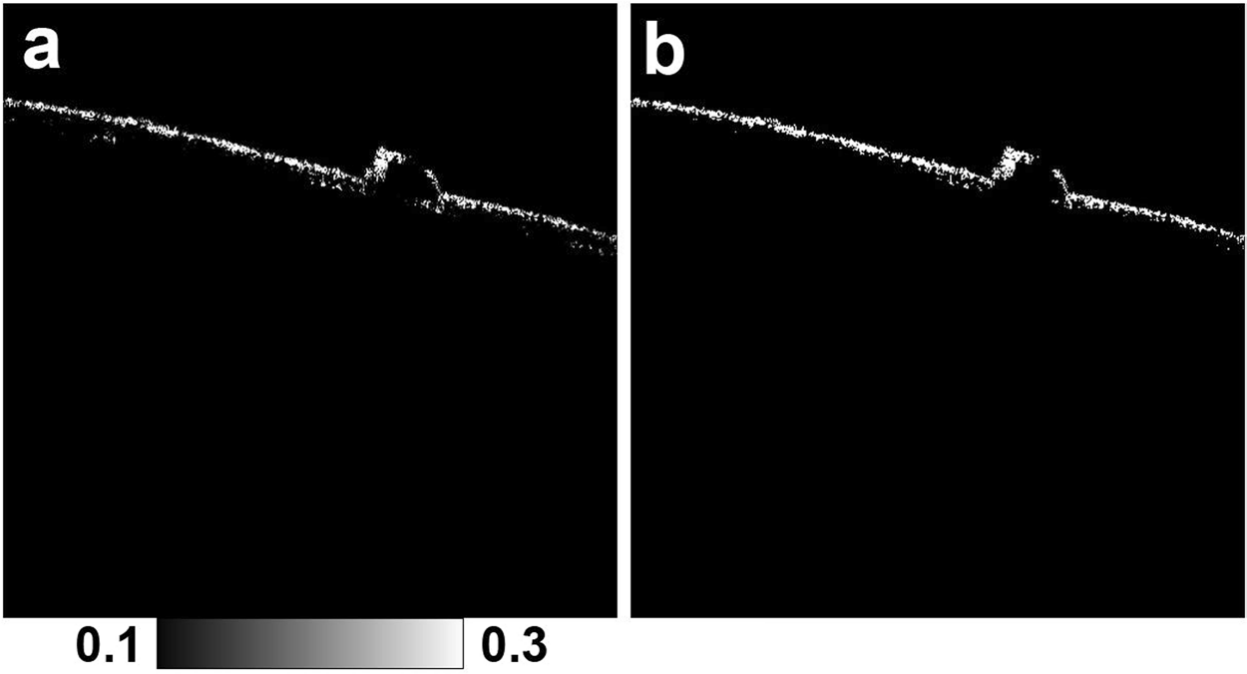
Image processing used to measure the area of focal RPE damage. (**a**) RPE-melanin cross-sectional image. (**b**) Binarized RPE-melanin cross-sectional image (F_RPE_ ≥ 0.15).

Hyper-transmission was defined as a localized stripe of increased intensity in the choroid beneath a PED in standard OCT B-scan images. The absence of scrolled RPE or other signs of an RPE tear were confirmed at those positions. Locations of hyper-transmission were surveyed with series of B-scan images by three ophthalmologists (M.M., S.U. and T.I.) after extensive discussion and analysis. The area (in mm^2^) of each point of focal RPE damage was calculated using the binarized RPE-melanin cross-sectional images (F_RPE_ ≥ 0.15, Fig. 8). The horizontal length of the defect of RPE melanin at each point of focal RPE damage was measured from the series of binarized RPE-melanin cross-sectional images. The total area of focal RPE damage was determined by summing the length of the defect in the individual B-scan images.

### Comparison with other clinical parameters

To evaluate the association of focal RPE damage with other clinical parameters, we compared its presence with the PED volume and HRF volume in PEDs. For the HRF volume, each standard OCT B-scan image was binarized using a Shanbhag method^40^, and the loci of HRF in the area of PED were manually selected in each binary image. Image processing software (Fiji; http://fiji.sc in the public domain)^41^ was used to measure the binarized areas of HRF^28^. The HRF volume in each eye was calculated by summing across the B-scan image series^28^. To calculate the PED volume, the inner boundary of the PED was manually selected in each B-scan image. Image processing software (Fiji)^41^ was used to measure the inner area of the PED, and the PED volume in each eye was determined by summing the volumes of individual segments using the Cavalieri principle of stereological analysis^42^.

## Supplementary information

Dataset 1
